# RTCB: an integrated deep learning model for garlic leaf disease identification

**DOI:** 10.3389/fpls.2025.1687300

**Published:** 2025-10-16

**Authors:** Jia Liu, Jingrun Kan, Xinjia Chen, Laixiang Xu, Xueli Zheng, Mohammad Nazir Ahmad, Junmin Zhao

**Affiliations:** ^1^ School of Computer and Data Science, Henan University of Urban Construction, Pingdingshan, China; ^2^ Faculty of Engineering, Science and Technology, Kuala Lumpur University of Science and Technology, Kuala Lumpur, Malaysia; ^3^ School of Digital Intelligence, Henan Xinxiang Vocational College of Industry and Commerce, Xinxiang, China; ^4^ Institute of Visual Informatics, Universiti Kebangsaan Malaysia, Bangi, Malaysia

**Keywords:** agricultural production, plant leaf disease detection, deep learning, improved ResNet18, attention mechanism

## Abstract

**Problem:**

Garlic is a common ingredient that not only enhances the flavor of dishes but also has various beneficial effects and functions for humans. However, its leaf diseases and pests have a serious impact on the growth and yield. Traditional plant leaf disease detection methods have shortcomings, such as high time consumption and low recognition accuracy.

**Methodology:**

As a result, we present a deep learning approach based on an upgraded ResNet18, triplet, convolutional block (RTCB) attention mechanism for recognizing garlic leaf diseases. First, we replace the convolutional layers in the residual block with partial convolutions based on the classic ResNet18 architecture to improve computational efficiency. Then, we introduce triplet attention after the first convolutional layer to enhance the model’s ability to focus on key features. Finally, we add a convolutional block attention mechanism after each residual layer to improve the model’s feature perception.

**Results:**

The experimental results demonstrate that the proposed model achieves a classification accuracy of 98.90%, which is superior to outstanding deep learning models such as Efficient-v2-B0, MobileOne-S0, OverLoCK-S, EfficientFormer, and MobileMamba. The proposed RTCB has a faster computation speed, higher recognition precision, and stronger generalization ability.

**Contribution:**

The proposed approach provides a scalable technical reference for the engineering application of automatic disease monitoring and control in intelligent agriculture. The current strategy is conducive to the deployment of edge computing equipment and has extensive significance and application potential in plant leaf disease detection.

## 1 Introduction

Crop diseases and pests are one of the main factors affecting plant growth and production. Crop diseases and pests can be detected and identified in time, and their management and control can be carried out effectively, reducing production losses and improving crop yield and quality. Leaves are an important basis for judging the degree of plant disease. Since most farmers do not have professional plant protection knowledge, it is difficult to implement the control plan (George et al., 2025). Machine vision technology, on the other hand, may detect leaf disease spots, assist in estimating disease severity, and advise farmers on how to take effective actions to maximize economic benefits.

Before the advent of the support vector machine (SVM) for feature vector classification, the primary method for extracting agricultural disease and pest features was manual. It also requires more data to generate feature vectors (Kalaiarasi et al., 2025). Feature extraction and classification techniques typically require segmenting the sick areas or leaves, even though they can have a higher recognition impact. It creates distinct feature extraction techniques for each condition, making it harder to differentiate identical diseases and increasing early-stage workload (Cai and He, 2021). Traditional machine learning-based disease detection methods must create distinct recognition models in addition to relying significantly on feature extraction.

Recently, deep learning technologies have advanced rapidly (Xu et al., 2021a), prompting attempts to apply machine vision to crop disease and insect pest recognition. For traditional machine vision algorithms (Cai et al., 2020), appropriate features must be selected in line with the target and prior knowledge (Goyal et al., 2025). These features usually include color, shape, and texture. The feature extractors are mainly manual designs. They are inconvenient and incapable of generalization. However, deep learning (Xu et al., 2021b) methods can adjust the weight parameters and build a suitable feature extractor. The process is relatively efficient and convenient. The feature extractors also have better generalization abilities, which can effectively overcome the shortcomings of traditional machine vision methods.

Although deep learning techniques have made significant progress in identifying plant leaf diseases, precise recognition of garlic leaf diseases still faces challenges in balancing model performance and efficiency. Although conventional deep learning models like the EfficientNet series have good recognition accuracy, their vast number of parameters and computational complexity limit their use in practical agricultural applications. Although lighter models such as ResNet18 have higher computational efficiency, they have shortcomings in feature extraction ability and key information perception. It leads to limited accuracy in identifying garlic leaf diseases in complex backgrounds. In addition, most current research is based on publicly available datasets with simple backgrounds and limited disease categories. The robust identification of garlic leaf diseases in complex backgrounds in real agricultural environments has not yet formed a good solution.

Based on the above challenges, we propose a lightweight model based on improved ResNet18. First, we utilized the triplet attention module to enhance the perception ability of key disease features. Second, we designed a CBAM to enhance feature expression ability. Finally, we used the PConv to reduce computational complexity. Our model achieves high recognition accuracy while keeping the parameter count low. It effectively solves the problem of balancing accuracy and efficiency in the recognition of garlic leaf diseases using lightweight models. The main contributions of this article are as follows:

1. A triplet attention mechanism is introduced to refine perceived information while preserving contextual information, effectively capturing key features in the data.
2. A CBAM dual attention mechanism is incorporated to enhance the model’s ability to express features and improve the network’s feature extraction.
3. A novel partial convolution network is designed to reduce parameter computation and improve model performance.

The remaining paper of this article is organized as follows: Section 2 analyzes the literature review of garlic leaf disease recognition technology. Section 3 mainly introduces the proposed method. Section 4 reports the experimental results and analyses. Finally, Section 5 presents the conclusion and future research directions.

## 2 Literature review

Machine learning techniques primarily use particular illness spot region segmentation to process the color, shape, and texture elements of the disease image (Shen et al., 2022). They are classified using a SVM classifier (Wang et al., 2022) and a normalized exponential function. For instance, Bala and Bansal (2024a) designed a model based on k-nearest neighbor (KNN), SVM, random forest, and Naive Bayes for pepper, potato, and tomato leaf disease classification and obtained an overall accuracy rate of 86.83%. Malik et al. (2024) designed a model based on CNN and SVM for corn leaf disease diagnosis and realized a classification accuracy of 99.8%. Li and Liao (2025) proposed a novel approach called support vector machines, graph cuts, and adversarial network for tea disease identification and reached an accuracy of 97.66%. Khan et al. (2024) provided a new method and compared random forest, XGBoost, GaussianNB, support vector machines, multinomial logistic regression, and KNN to classify tomato leaves and obtained an average precision of 98.527%. Kaur et al. (2024) reported a hybrid deep learning model based on a support vector machine, convolutional neural network, and convolutional block attention module for the early recognition and sorting of plant leaf diseases and reached an accuracy of about 98.72%. For traditional machine learning techniques, classification accuracy relies largely on human design. However, in certain complicated contexts with high noise levels, the picture recognition effect is subpar.

As artificial intelligence continues to develop, technologies for crop disease and pest identification have advanced in tandem. Crop pest and disease detection uses deep learning (Girmaw and Muluneh, 2024), transfer learning (Han and Guo, 2024), and reinforcement learning (Chelloug et al., 2023). Particularly, data-driven reasoning-based deep learning algorithms (Bala and Bansal, 2024b) enable rapid feature extraction from data, helping computer vision achieve higher accuracy and efficiency (Umar et al., 2024). For instance, Sharma et al. (2025) described a method for tomato leaf diseases based on the ResNet50, MobileNetV2, global average pooling2D, Batch Normalization, Dropout, and Dense layers that produced a precision of 99.92%. Goyal et al. (2024) developed a specific method for plant leaf disease identification, which relies on an optimized evolutionary gravitational neocognitron neural network. It achieved 99.92% and 99.98% accuracy when tested on two datasets. Ali et al. (2024) designed a lightweight deep learning model for apple leaf disease identification, achieving an accuracy of 98.6% and a classification rate of 98.25%. Mazumder et al. (2024) introduced a new architecture named DenseNet201Plus for banana and black gram leaf disease. This architecture includes preprocessing techniques, an attention-based transition mechanism, multiple attention modules, and dense blocks and achieved 90.12% accuracy on the banana leaf disease dataset and 99.50% on the black gram leaf disease dataset. Aldakheel et al. (2024) proposed an improved YOLOv4 model for automatic determination of plant leaf disease and used the Plant Village Dataset, which yielded an accuracy rate of 99.99%. Sahu et al. (2025) developed a hybrid model for multi-plant leaf disease classification. This model is based on a convolutional neural network deep learning architecture, and it showed an average accuracy rate of 97.36%. For the identification of various plant leaf diseases, Pan et al. (2024) proposed a convolutional neural network based on memristors. On two datasets—Plant Village and rice leaf disease—it produced identification accuracies of 99.03% and 99.16%, respectively. Xu et al. (2025) described an improved SPDNet and GrNet model for crop disease identification. It showed an overall classification accuracy rate of 98.96%. The majority of the test samples above are straightforward and have a single background, despite the fact that deep learning techniques have been used for crop picture detection. A lot of samples must be used to train the feature extraction capability.

## 3 Proposed methods

### 3.1 Triplet attention module

Based on the characteristics of garlic leaf disease data and considering the shortcomings of hardware and system performance, this paper initially chose ResNet18 for garlic leaf disease identification but found that the recognition effect was not ideal. Therefore, in order to improve the recognition accuracy of the model and more effectively capture key features in garlic leaf number data, we optimised the classic ResNet18 by introducing a triplet attention module after the first convolutional layer to enhance the network’s initial attention to features, preserve information context while refining perceived information, and improve the performance of classification tasks.

The triplet attention adopts a three-branch structure to capture cross-dimensional data features and interactively calculates attention weights on channels based on these features. The calculation process1.can be expressed as follows Equation 1.


(1) $$Z-pool(M)=[MaxPool_{0d}(M)ʘAvgPool_{0d}(M)]$$

where *MaxPool* is the maximum pooling operation, *AvgPool* represents the average pooling operation, ʘ is splicing operations, and 0d means the 0-th dimension for performing maximum pooling and average pooling operations.

For the input feature map $X\in R^{W\times H\times C}$ , pass it to the three branches of the triplet attention module. In the first branch, capture the cross-channel interaction features between spatial dimension H and channel dimension *C*. First, rotate *X* anticlockwise by 90° along the *H* axis to obtain $X_{H^{-}}\in R^{W\times H\times C}$ . Then, $X_{H^{-}}$ performs a Z-pool operation on the *W* dimension, performs a convolution operation, generates attention weights through the sigmoid activation function, dot multiplies the obtained attention weights $X_{H^{-}}$ , and rotates them clockwise by 90° along the *H* axis to obtain $X^{*}H$ , maintaining the original input state of *X*. The calculation process of the first branch is as follows Equation 2.


(2) $$X_{H}^{*}=(X_{H^{-}}\sigma {\left(\omega_{1}*\left(Z-\text{pool}\left(X_{H^{-}}\right)\right)\right)}_{H^{+}}$$

where *H^-^* is counterclockwise rotation of 90° along the *H* axis, *H^+^* represents clockwise rotation of 90° along the H axis, ω_1_ denotes convolution kernel, * is convolution operation, and σ represents activation function.

Similarly, in the second branch, the interaction between channel dimension *C* and spatial dimension *W* is captured. First, rotate *X* anticlockwise by 90° along the W axis to obtain *X_W -_* ∈*R^H×C×W^*. Then, *X_W_* performs the Z-pool operation on the *H* dimension, followed by the convolution operation. The attention weights are generated through the Sigmoid activation function, and the obtained attention weights are dot-multiplied with *X_W_*. Finally, *X_W_* is obtained by rotating clockwise 90° along the *W* axis while maintaining the original input state of *X*. The calculation can be expressed as follows Equation 3.


(3) $$X_{W^{-}}^{*}=\left(X_{W^{-}}\sigma \left(\omega_{2}*\left(Z-pool\left(X_{W^{-}}\right)\right)\right)\right){)}_{W^{+}}$$

where *W* ^-^ is counterclockwise rotation of 90° along the *W* axis, and *W^+^* represents clockwise rotation of 90° along the *W* axis.

For the third branch, the input feature *X* is reduced to 2 channels through the Z-pool operation and then convolved. The attention weight is generated through the sigmoid activation function, and the attention weight is dot multiplied with *X* to obtain the final feature result Equation 4.


(4) $$\begin{array}{c} X^{*}=X\sigma \left(\begin{array}{c} \omega_{3}*\left(Z-pool\left(\text{X}\right)\right) \end{array}\right) \end{array}$$

We will average the three components, and its mathematical expression is as follows Equation 5.


(5) $$X^{\prime }=\frac{1}{3}\left(X_{H}^{*}+X_{W}^{*}+X^{*}\right)$$

We input *X’* into a recurrent convolutional neural network. After convolution, we obtain a feature map as presented in the following equation Equation 6.


(6) $$V=\sigma \left(X^{\prime }\;\hat{*}\omega \right)$$

**Figure 1:**
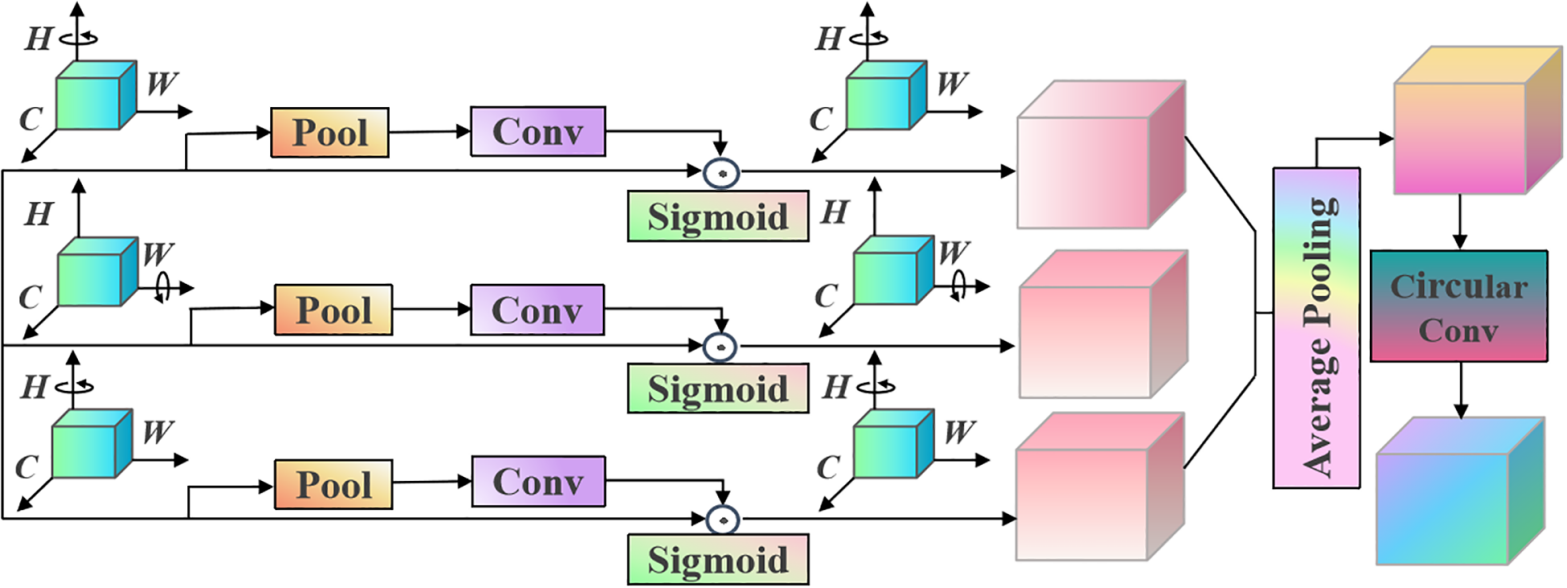
Proposed triplet attention structure.

The structure of the proposed recurrent convolutional neural network based on the triplet attention is provided in **Figure 1**.

The proposed triplet attention module captures cross-dimensional channel and spatial interaction information in a lightweight and efficient manner. It can enhance the model’s ability to focus on key features. The shallow features of the network usually contain rich detailed information (e.g., edges and textures), but not all details are equally important in garlic leaf disease recognition tasks. After the initial convolution, we immediately introduce the triplet attention module. It can screen and enhance the low-level features extracted initially, making the network more focused on potential areas related to diseases from the initial stage. It can provide higher-quality feature input for deep networks. Therefore, the proposed triplet attention module helps the model suppress background interference in the early stages and highlight key disease area features.

### 3.2 Convolutional block attention module

**Figure 2:**
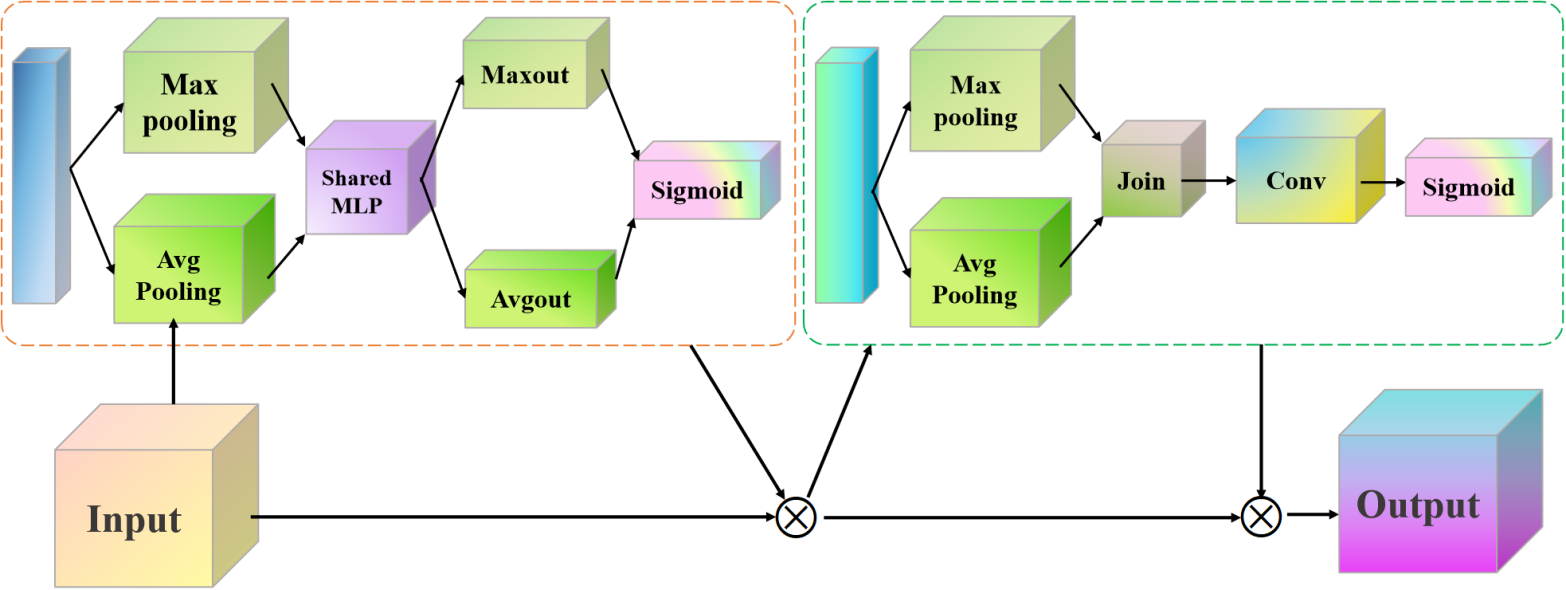
Proposed CBAM network architecture.

Although the proposed triplet attention module enhances the shallow network’s ability to extract detailed features, as the network structure deepens, the model still struggles to fully capture higher-order semantic features. In order to enhance the representation capability of deep networks, we embedded CBAM modules after the standard convolution operation of each residual block. The reason why we chose this position is that the features output by convolution need to be calibrated through the synergy of channel and spatial attention first. It can enhance discriminative features and suppress noise. It is then fused with the original input features through residual connections. This design method not only maintains the identity mapping property of ResNet to prevent gradient vanishing but also introduces an attention mechanism. It can optimize feature expression and maintain sensitivity to key features while deepening the network. The proposed CBAM structure is presented in **Figure 2**.

The proposed CBAM mainly consists of channel attention and spatial attention, which assign weights to each channel. It is usually operated using global average pooling (GAP) and global maximum pooling (GMP).

Assuming the input feature map *F* is *H×W×C*, the operational expressions of the CBAM for GMP and GAP can be defined as follows Equations 7, 8.


(7) $$F_{GAP}\left(c\right)=\frac{1}{H\times W}\sum_{i=1}^{H}\sum_{j=1}^{W}F\left(i,j,c\right)$$

(8) $$F_{GMP}\left(c\right)=\underset{i,j}{max}F\left(i,j,c\right)$$

Subsequently, the fully connected layer is used to learn the relationships between channels and obtain the corresponding weights. The goal of spatial attention is to assign a weight to each position in the feature map using a small convolutional kernel Equation 9.


(9) $$EM=\sigma \left(W_{k}*\left(F_{GAP}+F_{GMP}\right)\right)$$

where * is the convolution operation, $w_{k}$ means a convolution kernel, and σ is the Sigmoid activation function. Finally, we used these weights to update the original feature map.

### 3.3 Partial convolution

**Figure 3:**
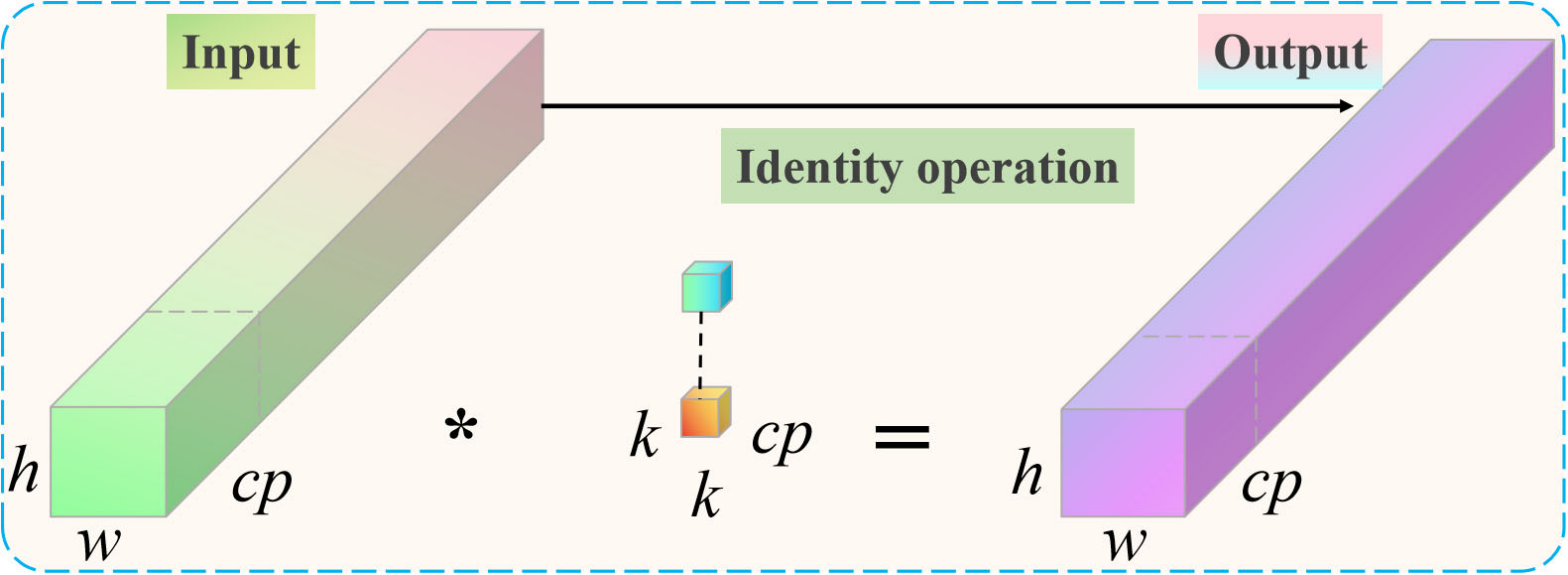
Proposed PConv convolutional architecture.

Although we added the triplet attention module and Convolutional block attention module to the optimized ResNet18 to improve the classification and recognition performance of garlic leaf diseases, it also increased additional computational overhead. In order to further construct an efficient and lightweight classification and recognition model, we designed a partial convolution (PConv) to reduce computational costs. PConv compresses the computational complexity and parameter count of the model by reducing redundant calculations. It only performs regular convolution operations on a portion of the input feature channels while keeping the remaining channels unchanged. We will replace the most computationally expensive 3×3 convolutional layer in the traditional ResNet18 residual block with a PConv layer. It can reduce the floating point operations (FLOPs) and parameter count (Params) of the model and significantly improve inference speed. This design approach makes the model more suitable for deployment on edge devices with limited computing resources while enhancing the representation capability of the original model. The proposed PConv convolutional architecture is presented in **Figure 3**.

For spatial feature extraction, the PConv only has to perform normal convolution on a subset of the input and output channels. The size of the remaining channels remains unaltered. The first or last continuous channel is regarded as the representative of the complete feature map for computation purposes when it comes to continuous or regular memory access. The number of channels in the input and output feature maps is equal without sacrificing generality. Compared to traditional convolution, PConv has a computational complexity that is only 1/16 of the former. The computational complexity *S* of PConv can be calculated as follows Equation 10.


(10) $$S=h\cdot w\cdot k^{2}\cdot c_{\text{p}}^{2}$$

where *h* is the height of the channel, *w* is the width of the channel, $c_{p}$ is the number of consecutive network channels, and *k* is the size of the filter.

**Figure 4:**
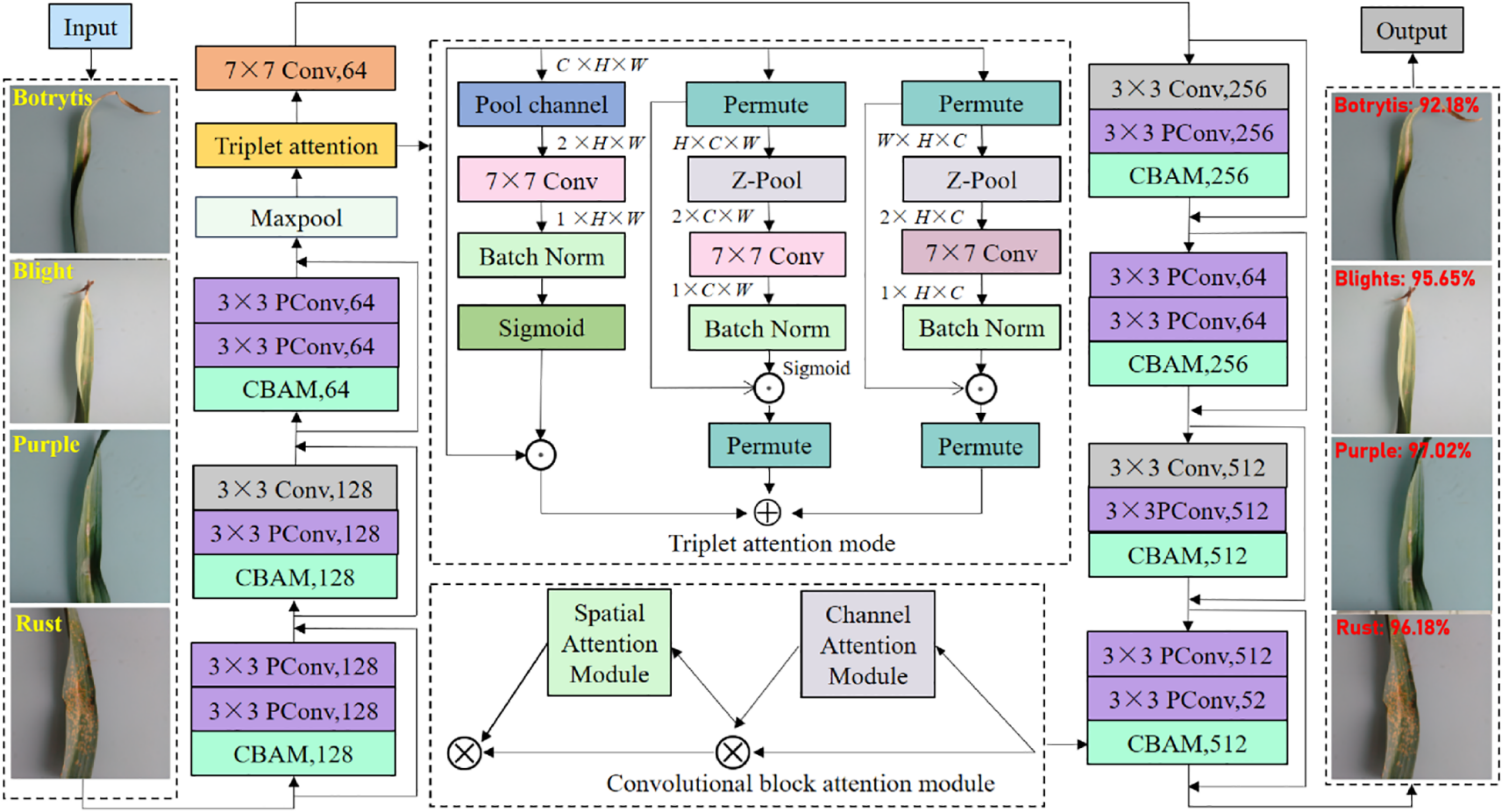
Proposed overall garlic leaf disease recognition framework.

Based on the above analysis, to visually demonstrate the overall recognition framework of the proposed garlic leaf diseases, we manifest it in **Figure 4**. First, we embed the triplet attention module after the first 7×7 convolutional layer of the traditional ResNet18. This location is in the shallow layer of the network, and the feature map contains rich texture, background, and contour features, but it also has a lot of noise. The triplet attention module interacts across dimensions. The triplet attention module simultaneously filters and enhances shallow features in both channel and spatial dimensions. It enables the model to focus on disease-related areas from the early stages of training, effectively suppressing background interference. It can provide more discriminative low-level feature representations for deep networks. Then, we add the CBAM module after the 3×3 convolution within each residual block of the traditional ResNet18. The CBAM module retains the identity mapping property of ResNet while achieving adaptive refinement of feature maps. It can effectively alleviate the problem of feature degradation in deep networks and enhance the model’s ability to extract high-order semantic features. Finally, we replace the standard 3×3 convolution in all residual blocks with the PConv. The PConv only performs convolution operations on some input channels, significantly reducing the number of parameters (Params) and FLOPs and greatly improving inference speed. This replacement significantly improves computational efficiency while maintaining the model’s representational ability. PConv can make the model more suitable for deployment on edge devices with limited computing resources. Through the above three designs, we have constructed a garlic leaf disease classification and recognition model that has both high recognition accuracy and high computational efficiency. Our proposed model not only enhances the ability to select and represent discriminative features but also achieves effective optimization of computational costs.

## 4 Experimental results and analysis

### 4.1 Experimental setup

The suggested network model is written in PyCharm 2024.2.3 with Python 3.9 and uses the deep learning framework PyTorch 2.3.1 along with the deep learning acceleration modules CUDA 11 and cuDNN 8.9 to speed up training. A 14-core Intel Ultra 5 processor with an NVIDIA GeForce RTX 4060 for acceleration makes up the hardware platform utilized for deep networks.

### 4.2 Data description

**Figure 5:**
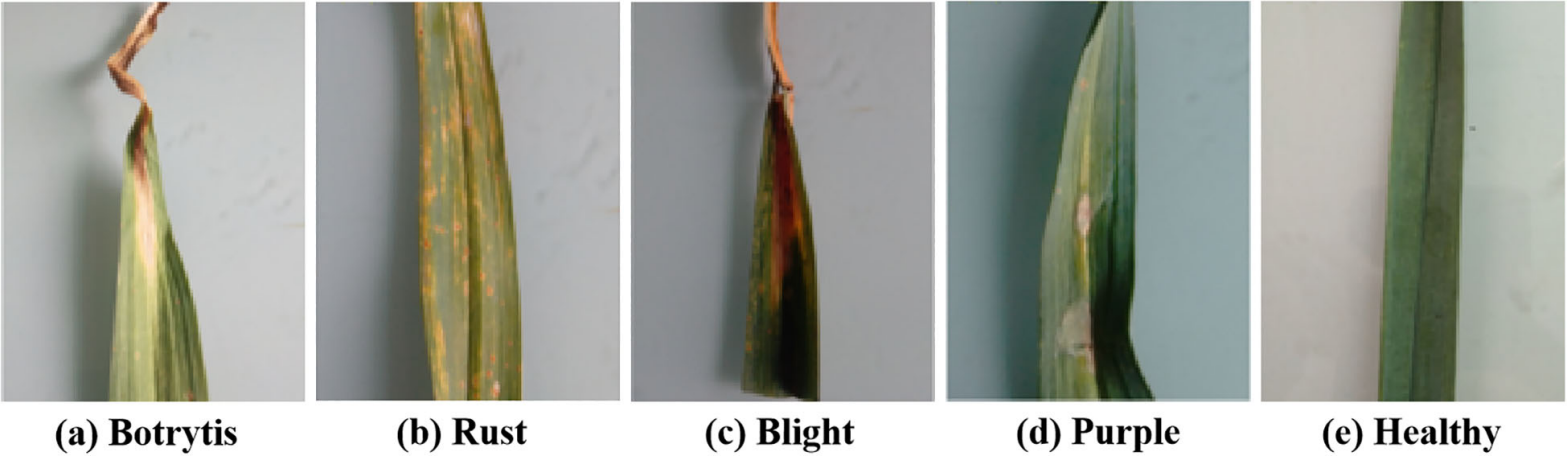
Experimental samples. **(a)** Botrytis, **(b)** Rust, **(c)** Blight, **(d)** Purple, **(e)** Healthy.

We obtained the data from the garlic planting bases in Henan Province, China. We used the mobile phone camera (Vivo S16e, China) to take the garlic leaves in a laboratory environment. The samples are shown in **Figure 5**.

There were 9076 samples in total, including healthy leaves and four types of diseases: rust, blight, purple, and botrytis. According to the division principle of most deep learning models, the 9076 samples were randomly divided into the training, validation, and testing sets with a ratio of 3:1:1. **Table 1** lists the detailed quantities of the samples.

**Table 1:** The specific distribution number of each type of data.

Categories	Training set	Validation set	Testing set	Total number
Botrytis	1177	392	392	1961
Rust	1124	374	375	1873
Blight	975	325	325	1625
Purple	1055	358	358	1771
Healthy	1108	369	369	1846
Total number	5439	1818	1819	9076

### 4.3 Train and test results

**Figure 6:**
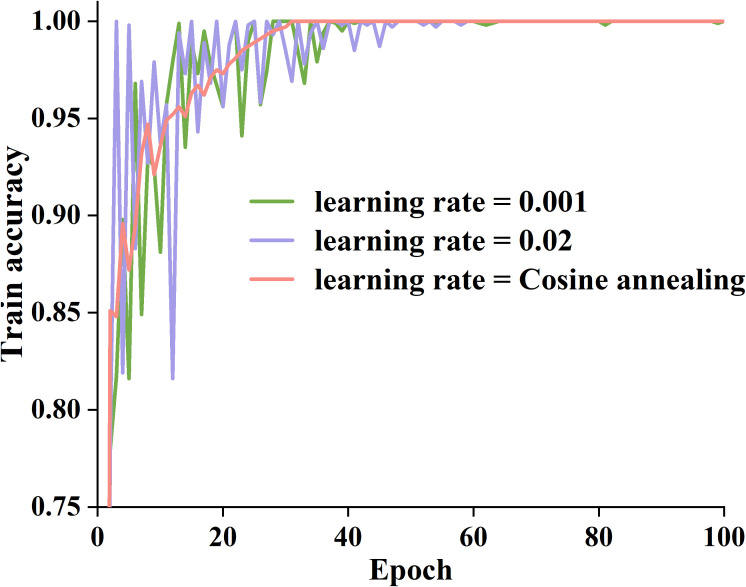
Training accuracy curves for different learning rates.

We used random search approaches to identify optimal training parameters for the proposed model, such as the activation function, learning rate, and network layers. We used learning rates of 0.001 and 0.02, along with a cosine annealing technique, to dynamically change the learning rate and assess the performance of the basic model. **Figure 6** presents three variations of training accuracy.

As can be seen in **Figure 6**, when the learning rate is 0.02, the model’s training accuracy rapidly increases to a higher level within 1~30 epochs, but there is a noticeable oscillation during the training process, which gradually stabilizes after 60 epochs. When the learning rate is 0.001, the model converges slowly, and the improvement in training accuracy is relatively small. In contrast, the cosine annealing strategy combines the advantages of both. After 30 epochs, the training accuracy of the cosine annealing strategy gradually stabilized, maintaining above 0.98, and there was no significant oscillation throughout the entire process. Therefore, we adopt a cosine annealing strategy to improve the training efficiency of the proposed model.

**Figure 7:**
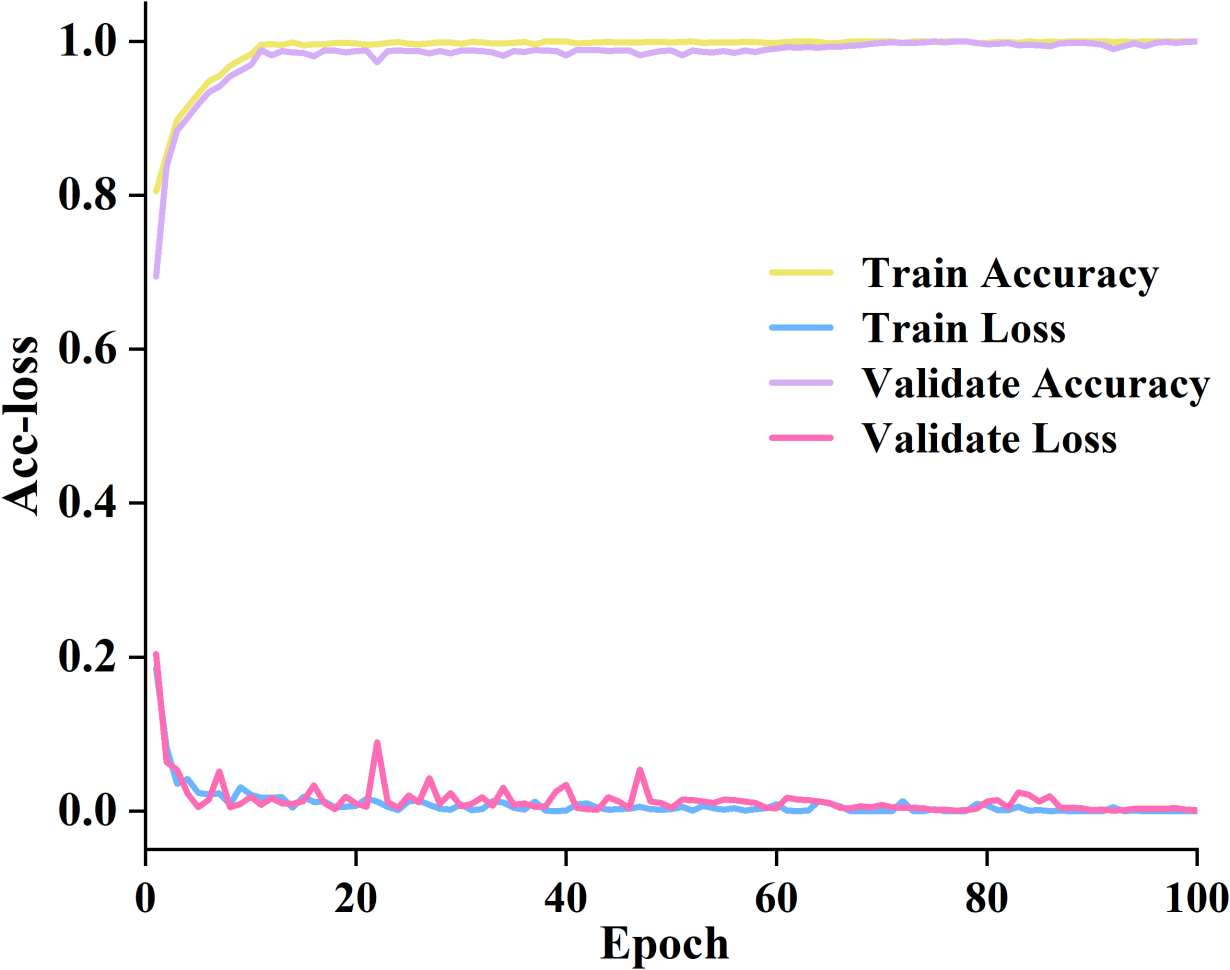
The accuracy and loss curves on the training and validation datasets.

Based on the selection principle of the above parameters, we trained the proposed model on the training set and validation set. The accuracy and loss value variation curves are manifested in **Figure 7**.

Detailed information has been acquired in **Figure 7**. Between 0 and 40 epochs, the training accuracy increased from 0.75% to 98.89%. The training loss decreased from 0.2 to nearly 0. The accuracy of verification is also improving, and the verification loss is also decreasing. Between 40 and 60 epochs, the training accuracy gradually stabilizes at around 98% with minimal fluctuations. The validation accuracy continues to approach the training accuracy. The verification loss has slight fluctuations but overall maintains a good level. It indicates that the proposed model fits well with the data. Between 60 and 100 epochs, all four curves tend to stabilize. It indicates that the proposed model has fully converged and achieved high and stable performance on both the training and validation sets.

**Figure 8:**
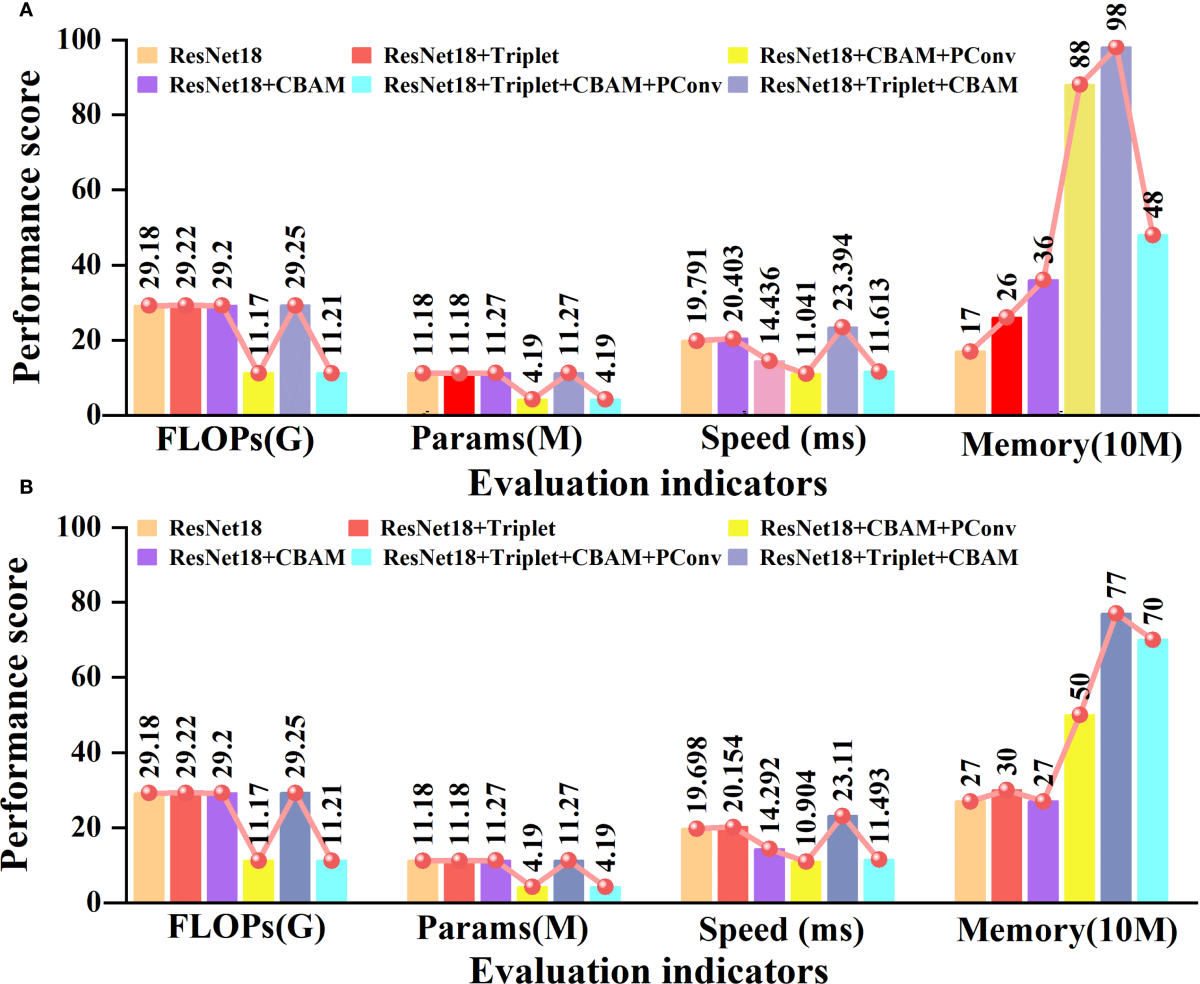
Ablation experiments of different evaluation indicators. **(A)** is the performance of our own garlic dataset. **(B)** is the performance of the open-source potato dataset.

To further validate the inference speed, robustness, and feasibility of edge deployment of the proposed model, we tested the parameters (Params), floating point operations (FLOPs), and running speed of five models in an environment with 32 g of memory and an Intel Core i5-13500H graphics card. The specific results are depicted in **Figure 8**.

Some important observations can be made from the content of **Figure 8**. In our own garlic dataset, adding the triplet attention module and CBAM module separately only slightly increased FLOPs and Params. When PConv is added separately, FLOPs and Params are reduced by 61.7% and 62.5%, compared to traditional ResNet18. In terms of inference speed, the ResNet18 with the CBAM added separately focuses on key features through an attention mechanism, which is 27% faster than traditional ResNet18. The memory usage slightly increases with the increase of modules, but the model incorporating PConv still has higher parameters and computational efficiency. On the open-source potato dataset, the impact trends of each module are not significantly different from those on the garlic dataset, indicating that the proposed model has good cross-dataset generalization ability. Overall, the model we propose can effectively balance computational complexity, parameter size, and inference speed. It has high potential for deploying edge devices in garlic leaf disease recognition tasks.

**Figure 9:**
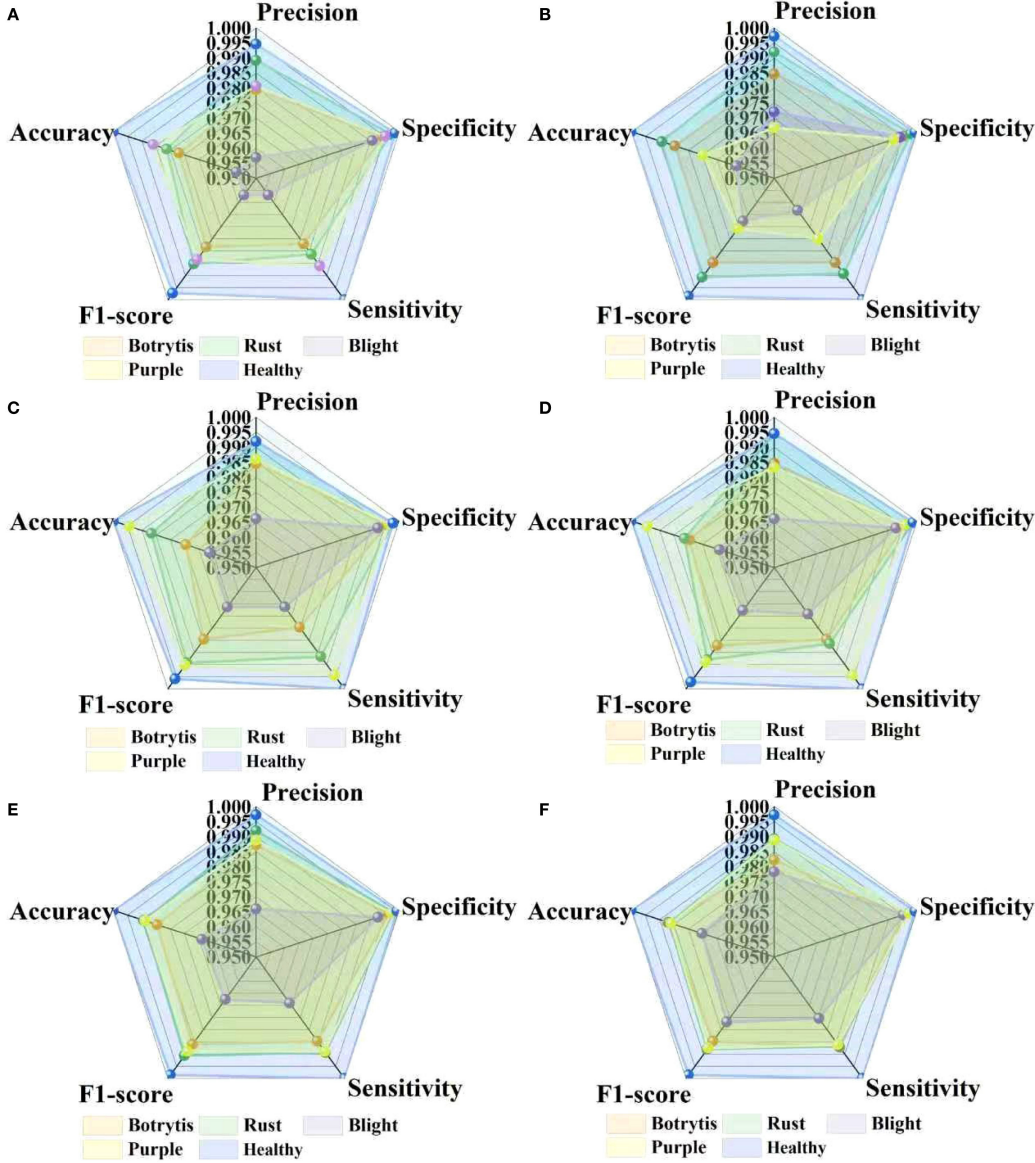
The ablation experiments of different indicators. **(A)** is classical ResNet 18. **(B)** is the ResNet 18+Trplet. **(C)** is ResNet 18+CBAM. **(D)** is ResNet 18+CBAM+PConv. **(E)** is the ResNet 18+CBAM+Trplet. **(F)** is the ResNet 18+CBAM+Triplet+PConv.

A radar chart is an efficient visual aid for demonstrating the relative benefits and downsides of various models in a variety of leaf disease classification tasks because it can graphically show several performance metrics in a single coordinate system. This visualization technique helps to highlight the model’s potential weaknesses for specific types of leaf diseases. To systematically assess the changes in diagnostic capacity of various models, we used radar plots to compare classification performance in **Figure 9**.

**Figure 9** illustrates that the proposed model outperforms the other five models in all five performance indicators. When the triplet attention is added, the model performs best on blind. When the CBAM module is added, the model performs best on the purple spot. Compared to the traditional ResNet18, its evaluation metrics have improved by over 8%. After introducing the PConv module on the basis of the CBAM, the model significantly reduces computational complexity while maintaining excellent classification and recognition performance. After adding the PConv, the rust disease outperforms the traditional ResNet18. The proposed ResNet18+CBM+Triplet combination model outperforms other individual modules in botrytis recognition. The three added modules do not conflict with each other. Overall, the proposed model has high classification performance for four garlic leaf diseases.

**Figure 10:**
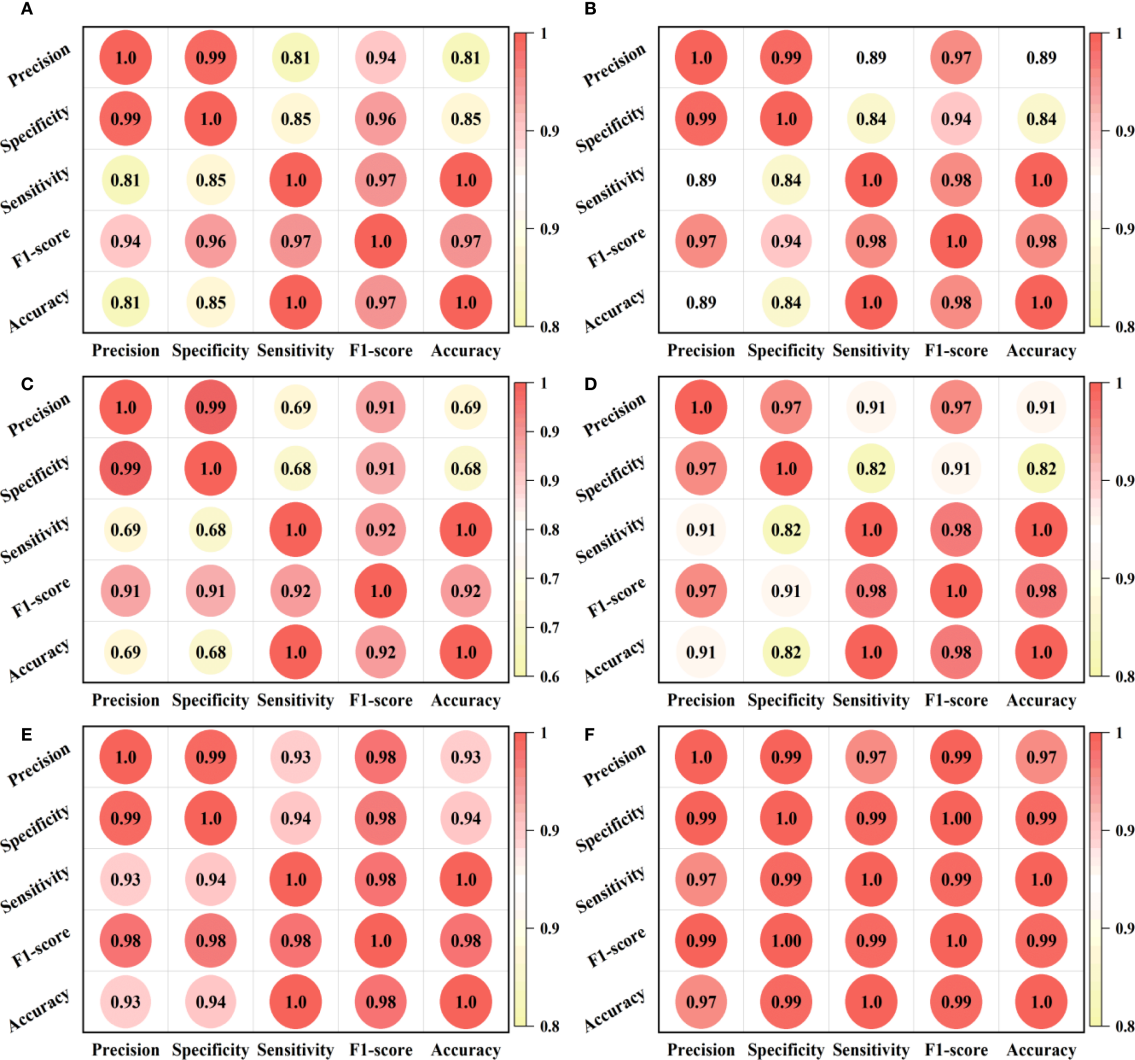
Heat map of the correlation between evaluation indicators of different models. **(A-F)** are the correlation heatmap analysis results of the classical ResNet18, classical ResNet18++Trplet, ResNet 18+CBAM, ResNet 18+CBAM+PConv, ResNet 18+CBAM+Trplet, the ResNet 18+CBAM+Triplet+PConv models, respectively.

The Pearson correlation coefficient heatmap is a matrix visualization method used to present the degree of linear correlation between variables in a dataset. The color depth of each cell in the figure corresponds to the Pearson correlation coefficient value of a variable pair (i.e., defined as [-1,1]), where the extreme value (i.e., ± 1) indicates complete correlation and 0 indicates no linear correlation. To explore the intrinsic connections between various performance indicators of the model and their synergy in multidimensional performance, we adopted this analysis method, and the results are presented in **Figure 10**.

From **Figure 10** we can see that the triplet attention module increases the correlation coefficient between evaluation indicators by 0.08 through feature fusion. The CBAM module may experience a decrease in correlation coefficient to 0.69 due to excessive focus on local features. After adding the PConv, the sparse computing mechanism effectively compensates for this deficiency, increasing the correlation coefficient by more than 0.05. When the CBAM is combined with Triplet, it exhibits more balanced performance. When three modules are added simultaneously, it exhibits a significant synergistic enhancement effect, with correlation coefficients exceeding 0.99 between most indicators. This confirms the feasibility of the multi-module deep fusion strategy.

**Figure 11:**
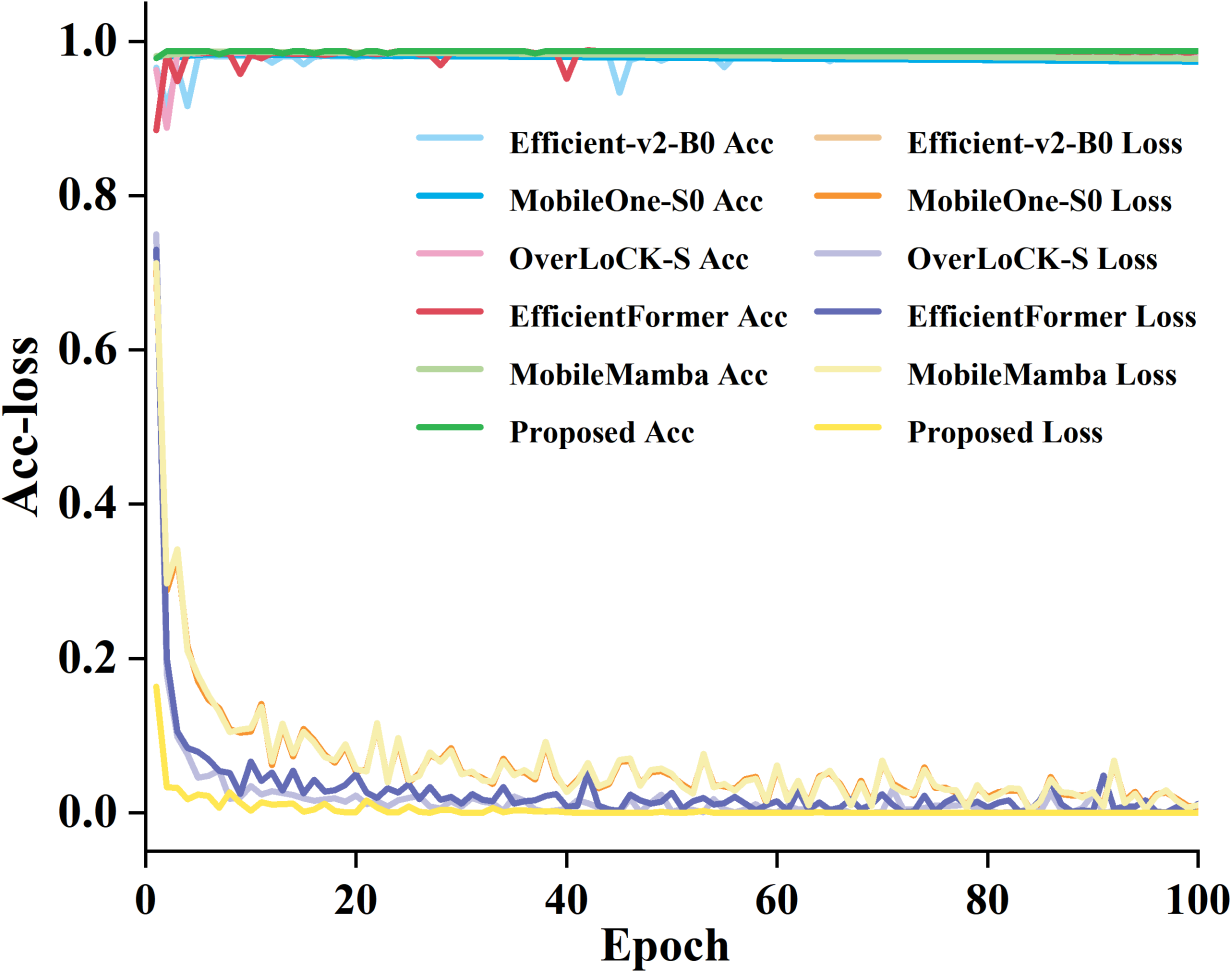
The training loss curves of the six models.

To compare the training accuracy and loss variation of the proposed model, we selected five common deep learning models, including Efficient-v2-B0, MobileOne-S0, OverLoCK-S, EfficientFormer, and MobileMamba, to train and compare with the proposed model on our dataset, as elucidated in **Figure 11**.

From **Figure 11**, it can be seen that the proposed model is superior to most mainstream lightweight models currently available. During the training process, the loss value of the proposed model gradually stabilizes. Although the model introduces attention mechanisms to increase complexity, PConv effectively compensates for computational overhead and maintains excellent performance while controlling parameters and computational complexity. Compared with Efficient-v2-B0 and MobileMamba, the proposed model not only has higher accuracy but also has a more stable training process and faster convergence. It indicates that the proposed model has more reliable performance in the task of identifying garlic leaf diseases while verifying the effectiveness and compatibility of the improved strategy.

**Figure 12:**
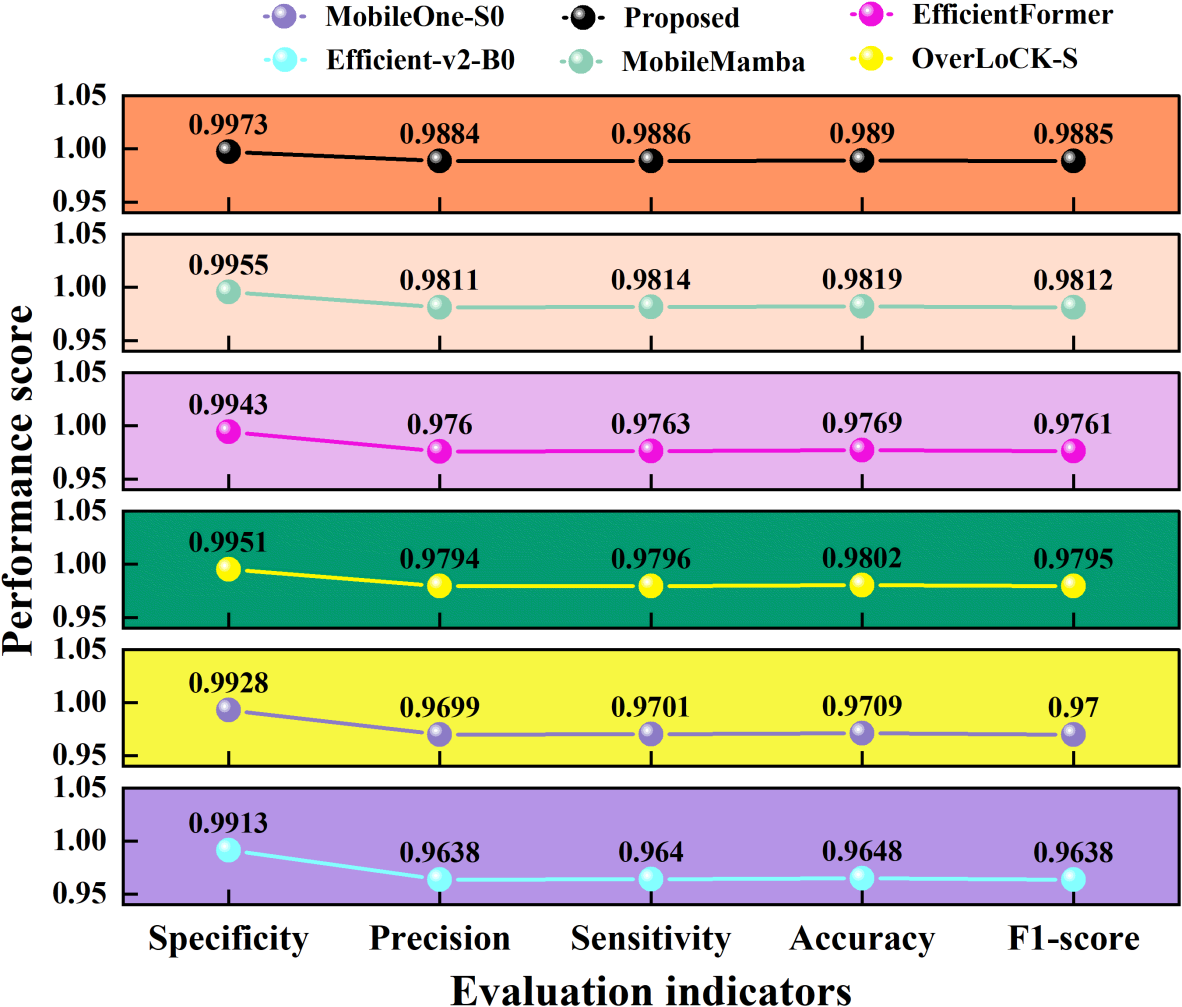
Performance scores of the six models.

We used specificity, precision, sensitivity, 1-score, and accuracy to evaluate the garlic leaf disease identification performance of different models, and the results are revealed in **Figure 12**.

The results from **Figure 12** indicate that the proposed model outperforms the other five excellent deep learning models in all five evaluation metrics. In terms of accuracy, the proposed model improves by 0.0241 compared to the lowest Efficient-v2-B0. Compared with the optimal MobileMamba, the proposed model improves by 0.007. In terms of F1 score, the proposed model improves by 0.0247 and 0.0073 compared to Efficient-v2-B0 and MobileMamba, respectively. Overall, the proposed model achieves bidirectional performance of lightweight and high precision in identifying garlic leaf diseases.

To more accurately present the specific performance of each model under different disease categories, we also counted the average accuracy of different models for four types of diseases. The results are presented in **Table 2**.

**Table 2:** Mean accuracy of different models under different diseases.

Methods	Botrytis	Rust	Blight	Purple
ResNet18	0.9703	0.9841	0.9705	0.9852
ResNet18+Triplet	0.9720	0.9857	0.9721	0.9735
ResNet18+CBAM	0.9832	0.9864	0.9738	0.9921
ResNet18+CBAM+PConv	0.9780	0.9864	0.9754	0.9936
ResNet18+CBAM+Triplet	0.9821	0.9900	0.9755	0.9858
ResNet18+CBAM+Triplet+Pconv	0.9848	0.9908	0.9807	0.9887

As can be seen in **Table 2**, the dual attention mechanism’s synergistic effect with PConv efficiently compensates for the inadequacies of previous models in feature association mining and complicated scene adaptation. The CBAM and triplet modules, whether introduced singly or in combination, can significantly increase classification performance. The proposed RTCB model achieves comparable average recognition accuracy for four garlic illnesses. It shows that the proposed model has the highest recognition stability. In summary, the suggested method employs a multi-module collaborative strategy and achieves excellent recognition performance. The proposed collaborative technique increases the accuracy and consistency of garlic leaf disease identification tasks. It demonstrates the rationale and practicality of the ablation module combo design.

To present the classification performance of the model for each category more intuitively, we further organized the total number (Tot), correct classification number (Cor), and incorrect classification number (Inc) of the five samples in the test set for the four models. The specific results are placed in **Table 3**.

**Table 3:** The correct and incorrect numbers of different models on the test set.

Method	Botrytis			Rust			Blight			Purple spot			Healthy		
	Tot	Cor	Inc	Tot	Cor	Inc	Tot	Cor	Inc	Tot	Cor	Inc	Tot	Cor	Inc
OverLoCK-S	392	386	6	375	371	4	325	312	13	358	346	12	369	368	1
EfficientFormer	392	384	8	375	369	6	325	311	14	358	345	13	369	368	1
MobileMamba	392	386	6	375	371	4	325	314	11	358	349	9	369	366	3
Proposed	392	390	2	375	373	2	325	318	7	358	350	8	369	368	1

**Table 3** reports that the overall classification accuracy of OverLoCK-S, EfficientFormer, MobileMamba, and the proposed model are 98.02%, 97.69%, 98.19%, and 98.90%, respectively. The overall classification performance of the proposed model is superior to the other three excellent deep learning models. Compared with the lowest EfficientFormer, the proposed model improves by 1.21%. The proposed model has the least number of misclassified samples for Botrytis and Rust. In the blink classification, there are many false positives of purple spots. This is because Bright and Purple Spot have certain similarities in the morphology and color characteristics of lesions. Overall, the proposed model has higher accuracy and stability in the classification of garlic leaf diseases, effectively reducing misjudgments and providing reference for precise identification and prevention of diseases.

**Figure 13:**
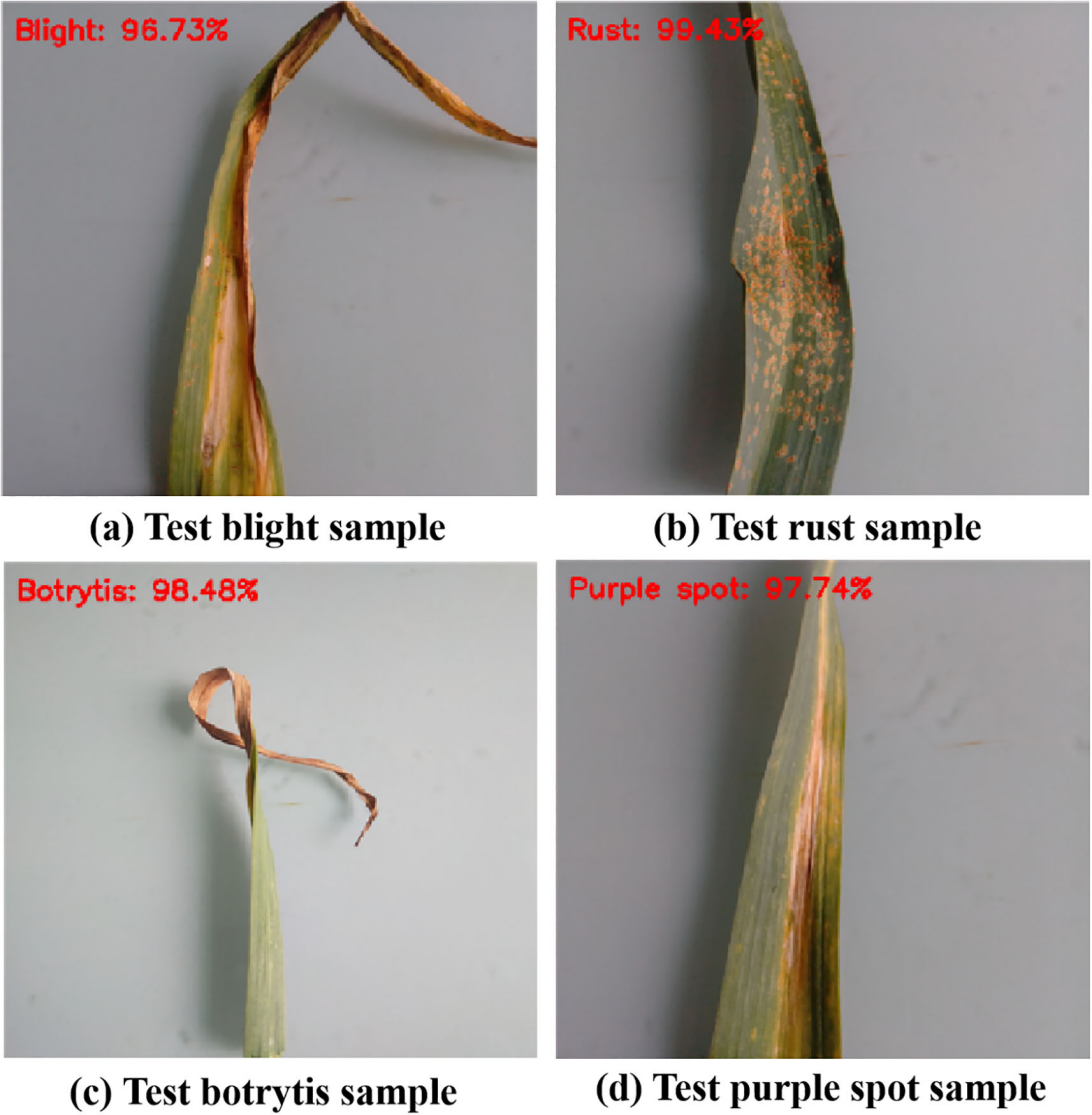
The predicted results. **(A)** Test blight leaf disease, **(B)** Test botrytis leaf disease, **(C)** Test rust leaf disease, **(D)** Test purple spot leaf disease.

We randomly selected a sample from each class in the test set for prediction, and the results are presented in **Figure 13**. All classifications are correct, and their recognition probability confidence is above 96%.

To verify the reliability of the model recognition at the individual level, we randomly selected a sample from each category in the test set for prediction. The visualization test results are revealed in **Figure 13**.

As shown in **Figure 13** that the proposed model can effectively capture the key features of different diseases. Even when facing samples with slight background interference and small lesion areas, it still maintains stable recognition ability. This confirms that the proposed model has high reliability in single-sample classification and can provide effective technical support for precise identification of individual plant diseases in the field. Therefore, our proposed model not only helps to achieve automated classification and recognition of garlic leaf diseases but also provides a scalable solution for the development of lightweight intelligent agricultural systems. It further confirms that our proposed strategy has strong application potential and promotional value.

### 4.4 Deployment potential on edge devices

To evaluate the applicability of our proposed model in practical agricultural environments, we analyzed its deployment potential on edge devices. We focus on analyzing the lighting changes, complex field backgrounds, and inference performance of the model in real-world scenarios with limited resources on smartphones.

In Section 4.3, we introduced PConv to reduce the parameter count to 4.19M and floating-point computation to 11.19G while maintaining high accuracy. Compared with the traditional ResNet18, this efficiency has been improved by 60%. This lightweight design significantly reduces the demand for memory and computing resources, laying a solid foundation for deploying the model on edge terminals such as smartphones, embedded devices, or agricultural drones.

The dataset we used was mainly collected in laboratory environments, but during the training process, we perform random brightness adjustment, contrast variation, noise injection, and simulated shadow processing on the data. These data augmentation methods can enhance the model’s adaptability to changes in lighting and complex field conditions. During the training process, the model gradually approached stable convergence without any significant oscillations. This indicates that the model has high robustness to changes in the input image. In addition, the experimental results verified that the proposed model maintains high stability in identifying garlic leaf diseases with high accuracy. The accurate classification with high confidence further proves that the model can effectively cope with intra-class differences and has the preliminary stability required for practical applications.

To verify the actual reasoning ability of the model on the smartphone end, we converted the trained model into ONNX format files and integrated them into an Android prototype application based on PyTorch Mobile. The testing equipment uses Xiaomi 13 (Snapdragon 8 Gen 2 processor, China). The inference test for a single image shows that the average inference time of the model is about 55 ms, and the peak memory occupation is about 50 MB. This test result indicates that the model can achieve image recognition on mainstream smartphone terminals, meeting the real-time diagnosis needs in the field. Although there are some differences between the current testing environment and real field conditions (e.g., strong lighting, multiple occlusions, and complex backgrounds), the existing performance has provided a reliable basis for subsequent field testing and system optimization.

In future research, the application scenarios of our proposed RTCB model will be further expanded to diverse edge devices. Considering the limitations of edge computing resources on high-throughput inference, we plan to optimize image preprocessing and data transmission protocols for batch images collected during low-altitude drone flights. We will upload the real-time image data collected by the drone to the cloud server. We will use the RTCB model deployed in the cloud to achieve plant leaf disease detection. At the same time, by combining the drone path planning algorithm with the real-time feedback mechanism of cloud detection results, we will dynamically adjust the drone flight trajectory to achieve rapid screening of diseases in 10000 acres of garlic fields. We plan to use knowledge distillation technology for portable detection terminals used by grassroots agricultural technicians. We use the proposed RTCB model as a teacher model to train a lightweight student model. It meets the terminal memory limit while ensuring low accuracy loss. To support the analysis of regional disease trends, we will also build a collaborative architecture of the edge cloud. We use portable terminal edge devices to locally store key diagnostic results. We upload it to the cloud management platform using the 5G network. This strategy avoids bandwidth consumption caused by a huge quantity of image transfer, resulting in efficient data synchronization between edge devices and the cloud.

Although we have made some progress in model lightweighting and inference efficiency, we still face multiple challenges in the actual deployment process. Firstly, the currently proposed model is mainly trained based on images collected in laboratory environments. The real field environment has extreme lighting conditions, complex background interference, and multiple occlusions. These factors may all affect the recognition performance of the model. Therefore, in the future, we will systematically collect field disease images covering different meteorological conditions, crop growth periods, and shooting angles to construct a more representative and diverse dataset. Meanwhile, we plan to introduce domain adaptation techniques to enhance the model’s generalization ability across environmental scenarios. Secondly, in order to further adapt to low-end devices with limited computing power, we will use INT8 quantization and knowledge distillation compression techniques to reduce model size and improve inference speed. Finally, practical applications require not only efficient recognition algorithms but also integrated image preprocessing, result interpretation, user interaction, and data communication modules. Therefore, we will coordinate and optimize power consumption, memory management, and response time at the system level and build an end-to-end intelligent agricultural diagnostic solution.

## 5 Discussion

In recent years, deep learning has demonstrated substantial effectiveness in the classification of plant leaf diseases. Yao et al. (2024) introduced a leaf disease recognition system with an integrated enhanced attention module, which achieved a 93.64% accuracy by autonomously learning and extracting important information from lesions. Although it enhanced the algorithm’s overall recognition performance for each leaf disease, the accuracy was slightly lower than that of better-performing models. Bera et al. (2024) suggested an attention-based method for plant disease classification networks. This method achieved 97.74% accuracy on the PlantPathology dataset. However, it focuses on key regions of leaves and has certain flaws in global feature collection. Similarly, an enhanced CNN model was suggested by Li et al. (2024). It can classify and recognize healthy chili leaf images as well as images of chili leaves with four different pathologies, and it obtained an accuracy of 93.5%. Although it improved the model’s ability to extract basic aspects of chili leaf illnesses, it was unable to acquire comprehensive information. As a result, the efficacy of discriminating between the general symptoms of leaf curl illness and yellowing disease is slightly reduced. This article differs from previous research in that it not only optimizes the structure of ResNet18 to improve the depth of feature extraction, but it also introduces a CBMA model and a triplet attention model, resulting in comprehensive capture of both local and global features. Through ablation tests, we established an important finding. When the CBMA and triplet attention modules are combined, they boost identification accuracy far more than a single attention mechanism does. This shows that their fusion produces a synergistic effect.

Nonetheless, there are certain limitations, even if the approach described in this article has greatly improved recognition accuracy. First and foremost, it remains to be seen whether the current dataset can detect a wider range of garlic leaf disorders, given it only comprises three common disease types. Second, the model’s comprehensiveness and utility in real-world applications are limited because it is currently insufficient for measuring illness severity and progression.

These limitations mainly come from two aspects. First, the dataset lacks sufficient diversity and complexity. Second, there is room to improve the model’s understanding of advanced semantic information—specifically during the processes of feature extraction and classification decision-making. To solve these problems, future research has two main directions. One is to expand the dataset so it covers more types of garlic leaf disease images. The other is to study multimodal data fusion techniques, such as fusing image, spectral, and environmental data. Both directions aim to enhance the accuracy and reliability of disease recognition. Meanwhile, model performance might be enhanced by refining its structure and integrating more complex attention processes or advanced technologies like graph neural networks. These actions enable the model to better capture the intricate interactions between illness features, which in turn benefits its performance.

Through this research, we have gained a thorough understanding of the immense potential of deep learning in intelligent agricultural management, but we have also identified the inadequacies of present models in practical applications. As a result, we suggest a new hypothesis: by including time series data and growth cycle information, deep learning models may not only detect the types of garlic leaf illnesses, but also define the period during which they occur. Based on this premise, we propose that future study focuses on the dataset’s diversity and complexity. By collecting image data of garlic leaves at various growth and disease development stages, a dataset incorporating time series information can be created, providing a more refined solution for agricultural disease monitoring. Meanwhile, we will continue to simplify the model structure, minimize computational complexity, and optimize it for edge computing devices. Terminal devices such as drones and portable detectors can be used for integration to develop lightweight chips that fit garden drones. This integration not only makes the practical edge application of plant disease and pest identification systems possible but also provides backing for the long-term development of garden plant health monitoring systems.

## 6 Conclusions

In this work, we propose an integrated triplet attention, CBAM dual attention mechanism, and partially convolutional deep learning garlic leaf disease classification and recognition model, and achieve a classification and recognition accuracy of 99.92% on our test set. This proves that the currently proposed model has great potential for development in the field of garlic leaf disease identification.

The proposed research has yielded significant findings, but it also has limitations. First, the proposed model was only applicable to identifying healthy garlic, botrytis, rust, blight, and purple disease types. Second, it is not suitable for identifying multiple diseases on one leaf. Third, the number of garlic disease samples we collected is not enough, and the types of garlic leaf diseases are not comprehensive. Last, the identification accuracy is not ideal with different meteorological conditions, shooting angles, and degrees of damage.

Future work still needs to focus on data augmentation, model optimization, and real-time performance improvement to address practical challenges such as extreme weather and garlic leaf disease occlusion and further promote the development of intelligent transportation systems. We will conduct research in the following areas:

Although the proposed system has achieved good results in the identification of garlic leaf pests and diseases, there are still many shortcomings that need further research and improvement. The specific plans are as follows:

1. Diversification of dataset: We will collect and integrate garlic leaf data from different regions and environments to build a more comprehensive and diverse database. This will help improve the generalization ability of the model in practical applications.
2. Dataset diversification: We will create a large-scale dataset that combines multimodal data (e.g., Pictures, spectra, and environmental data), encompassing several garlic production sites, diverse planting settings, and full development cycles, as well as disease severity classification. To eliminate distributional discrepancies, we will apply data augmentation and domain adaptation approaches (e.g., DANN). We will evaluate the model in a variety of illumination, weather, and planting modes. We will create a dual scenario assessment system from the lab to the field and ensure practical reliability.
3. Implementing dynamic monitoring and functional upgrades: To meet actual monitoring needs, we will upgrade from static image processing to real-time video stream analysis. The goal is to develop a function that can continuously monitor bean planting areas, achieve automatic real-time identification and data storage of pests and diseases, significantly improve the timeliness, practicality, and response speed of the system, and provide strong support for timely prevention and control.
4. Real-time performance optimization: In response to the real-time requirements in practical applications, we will optimize the model structure and algorithms, improve data processing efficiency, reduce parameter calculation complexity, and adapt to more real-time field scenarios.

## Data Availability

The original contributions presented in the study are included in the article/supplementary material, further inquiries can be directed to the corresponding author/s.
